# Editorial: Diagnostic Procedures in Veterinary Microbiology and Infectious Diseases

**DOI:** 10.3389/fvets.2022.868741

**Published:** 2022-02-25

**Authors:** Valentina Stefanetti, Doreene Hyatt, Fabrizio Passamonti

**Affiliations:** ^1^Department of Veterinary Medicine, University of Perugia, Perugia, Italy; ^2^Department of Microbiology, Immunology and Pathology, Colorado State University, Fort Collins, CO, United States

**Keywords:** veterinary microbiology, veterinary infectious diseases, novel diagnostic technique, veterinary diagnostic laboratories, diagnostic assays

The authors are pleased to have been invited to serve as Guest Editors for the Research Topic entitled: “Diagnostic Procedures in Veterinary Microbiology and Infectious Diseases.” This Research Topic is a collection of 19 manuscripts (14 original research papers, four brief research reports, and one mini-review article) published from 130 authors, which, so far, have received thousands of views. The geographical areas represented include Europe (*n* = 6), North America (*n* = 5), South America (*n* = 1), Australia (*n* = 1), and Asia (*n* = 6).

Infectious diseases are relevant to veterinary medicine, both in companion animals and in livestock, due to their ability to induce clinical illness and/or economic losses. Additionally, infectious diseases of animals are relevant to public health in that the causative organisms can be carried by animals and be spread to humans to cause zoonotic disease. Knowledge of the pathological mechanisms that microorganisms have developed is a critical issue for a correct and prompt diagnosis, which can be life-saving for animals. Inspired by the recent SARS-CoV-2 pandemic, we thought about the importance of working from the One-Health perspective and about how timely diagnosis of infectious diseases represents a significant challenge.

Diagnosticians use two primary methods to detect infectious diseases: confirming the presence of the microorganism or detecting antibodies against the organism. Despite the availability of an array of techniques, among the last-generation assays, no single test is usually considered the definitive golden standard, and that is the reason why there is still an urgent need for research on infectious disease diagnosis.

Therefore, out of the various research questions proposed by manuscripts submitted in this Research Topic section, the large majority of contributing studies describe new advances, i.e., novel assays or improvement of pre-existing tests for diagnostic purposes, in veterinary microbiology for several animal species.

Pig production is one of the fastest growing livestock sectors in the world. That is probably the reason why several contributions in our Research Topic are related to the main infectious diseases, which represent a significant threat for the swine industry worldwide. Of note, African swine fever (ASF) has caused great economic losses in the pork industry, and the current lack of commercially available vaccines makes the prevention and control of ASF worldwide even more challenging. Yu et al., described the production and characterization of p30 monoclonal antibodies and the subsequent development of an ELISA kit for early detection of ASF virus (ASFV). This kit has high diagnostic specificity and sensitivity and is able to detect seroconversion in infected pigs as early as 10 days post-infection (dpi). Therefore, this assay is a very useful tool for early detection of ASFV. Jia et al., described another method for ASFV DNA quantification, which utilizes a next-generation PCR platform, nanofluidic chip digital PCR. Due to its high sensitivity, this assay appears to be very useful, especially for the recognition of early ASFV infection. Velazques-Salinas et al., in an attempt to find a potential vaccine to protect pigs against ASFV, developed attenuated vaccine candidates by deleting critical viral genes associated to virulence. In their paper, they describe how they developed three new quantitative PCR (qPCR) assays for differentiation between infected and vaccinated animals (DIVA). Collectively, the results of this study demonstrate the potential of qPCR assays as tests supporting ASFV vaccination.

Among other porcine viruses explored in our collection, Mai et al., investigated the high coinfection status of novel porcine parvovirus 7 (PPV7). Sows with porcine circovirus 3 (PCV3) experience reproductive failure, suggesting that PPV7 may play a role in infection as cofactor by enhancing PCV3 replication. More analyses of the exact PPV7 pathogenesis mechanism are warranted, and there is an urgent need to further investigate coinfections in the porcine field. Liu et al., studied porcine astroviruses (PAstVs), which are prevalent in pigs and of which five genotypes have been reported worldwide. Multiple PAstV genotypes and coinfection and genetic recombination events have often been reported. However, there is still no diagnostic method available for PAstV genotype detection. In this study, a multiplex reverse-transcription PCR (RT-PCR) method, which is efficient and convenient for the diagnosis of five known PAstV genotypes, was established. This method is a valuable tool for the differential diagnosis of PAstVs circulating in pig herds and will facilitate the surveillance of PAstV coinfection.

The emergence of new viruses or variants due to spillover or mutation or recombination events makes monitoring of porcine enteric coronavirus (CoV) of utmost importance in order to curtail their spread and allow for updated diagnostic tools. The emergence of new CoVs and the re-emergence of porcine epidemic diarrhea virus (PEDV) worldwide require studies characterizing these CoVs on pig farms in order to allow an accurate differential diagnosis of viral diarrhea. Although Puente et al., demonstrated that PEDV is the only CoV currently circulating in Spain, they highlighted the need for constantly monitoring porcine enteric CoVs in order to prevent their spread and allow for updated diagnostic tools. So, gastrointestinal infectious diseases affect pigs worldwide and are one of the major concerns in many countries. The disease occurs usually at the post-weaning stage and impairs pig performance, weakens welfare, and causes economic losses to farmers due to medication costs as well as slow growth. One of the main challenges to investigate the health status of pigs is to find a less stressful and non-invasive sampling method. Sali et al., explored the dynamics of salivary biomarkers such as adenosine deaminase (ADA), haptoglobin (Hp), and cortisol from saliva samples of growing pigs exposed to LPS challenge. Their study suggests that ADA and Hp can be used as inflammatory biomarkers in pigs.

Infectious diseases of livestock are a major threat to global animal health and welfare and their effective diagnosis is crucial also for human health. Okda et al., developed diagnostic tools to differentiate between influenza D virus (IDV), a novel orthomyxovirus emerging in cattle worldwide, and the human influenza C virus (ICV), through an ELISA test. It is critical to develop diagnostic tools and assays to differentiate between ICV and IDV due to their similar genomic structures. These authors successfully developed a blocking ELISA assay able to differentiate between these two closely related viruses.

Bovine respiratory disease (BRD) is one of the major causes of losses for the cattle industry worldwide, and the efforts to improve diagnostic procedures to identify pathogens caused BRD are remarkably increasing, also to provide some indication for veterinarians. Klompmaker et al., used high-throughput real-time PCR to detect some bovine respiratory pathogens and quantified the cut-off values for pathogens associated with BRD in an effort to analyze the association between these findings and clinical observations. The authors found that it is possible to suggest clinically relevant cut-off values with statistically significant associations with clinical scores indicating respiratory disease for *P. multocida, M. bovis*, and *H. somni*. This study thereby presented an interesting approach for objective and veterinary field-relevant diagnostic test interpretation. The same research group, in the wider project of Goecke et al., aimed to design and develop a high-throughput real-time PCR system for the detection of significant respiratory and enteric viral and bacterial bovine pathogens. Nowadays, detection of these pathogens is costly and time consuming due to the methodology used and due to the fact that several different PCR assays are needed to cover the wide range of circulating pathogens. Their study shows that the high-throughput real-time PCR method allows simultaneous analysis of a large number of samples and contributes to more detailed diagnostics.

Another biomolecular assay was developed by Hole et al., for the diagnosis of vesicular stomatitis virus (VSV). Vesicular stomatitis virus causes a disease in susceptible livestock that is clinically indistinguishable from foot-and-mouth disease (FMD). Rapid testing is therefore critical to identify VSV and rule out FMD. The authors improved a previously published multiplex real-time RT-PCR (mRRT-PCR) assay. In this paper they highlight the challenges that the large genetic variability of VSV poses for virus detection by mRRT-PCR and show the importance of frequent re-evaluation and validation of diagnostic assays for VSV to ensure high sensitivity and specificity. Similarly, Subbiah et al., focused on the emerging field of next-generation sequencing and metagenomics to address the problem of detecting disease conditions in veterinary science with a metagenomic shotgun sequencing approach for unbiased detection of the microbiome. The authors used horses that were suspected to have tick-borne diseases and demonstrated the detection of *A. phagocytophilum*, suggesting that this approach can be used to detect blood-borne pathogens.

In recent years, the incidence of brucellosis has increased annually, causing economic losses in various countries. Therefore, the development of rapid, sensitive, and specific diagnostic techniques for brucellosis has become critical. Yin et al., used “immunoinformatic” technology to predict the B cell epitopes in the major outer membrane proteins of *Brucella*, establishing an ELISA method for brucellosis diagnosis based on a multi-epitope fusion protein that can be used to assess the serum of bovine, goats, and other livestock.

One of the aims of our Research Topic section was to find new diagnostic approaches to replace the so-called golden standard tests. Dall'Ara et al., demonstrated the utility of an in-clinic ELISA test in detecting protective antibodies against canine parvovirus (CPV) in adult dogs and compared it with the golden standard, the hemagglutination inhibition (HI) test, both after vaccination and/or infection. Moreover, in unvaccinated puppies, specific CPV maternally derived antibody titers can be measured more easily by an in-clinics ELISA test, allowing the prediction of the best time of vaccination, thus reducing the rate of vaccination failures.

Silvestri et al., investigated the methicillin resistance of *S. pseudintermedius* (*SP*) through a new approach. The resistance is mediated by the *mecA* gene, which encodes the PBP2a protein and conveys resistance to β-lactams. The authors showed that immunofluorescence is a promising technique, with a good capability of correctly identifying resistant and sensitive *SP*. Immunofluorescence has the potential to be applied as a screening method, independent from the golden standard, bacterial culture. Therefore, it represents a new interesting tool for both research and diagnostics.

Guedes et al., investigated the usefulness of the ranking technique to predict the most likely infecting serogroup of *Leptospira*. The microscopic agglutination test (MAT) used for the serological diagnosis of leptospirosis, as a robust and inexpensive method, is still the reality in many laboratories worldwide. However, both the performance and the interpretation of the MAT vary from region to region, making standardization difficult. Of course, MAT replacement may be difficult, but the authors proposed the ranking technique as another way of interpreting the results obtained by MAT, in order to refine the data and reduce the occurrence of cross-reactions between the serogroups, thus demonstrating that this technique can be useful in the MAT for predicting the most likely infecting serogroup of *Leptospira* and can be applied in epidemiological studies involving herds.

Hwang et al., performed an interesting study on chronic wasting disease (CWD), which is a transmissible spongiform encephalopathy in free-ranging and captive cervid species. The disease process is ultimately fatal and occurs through the misfolding of a normally occurring protein. Detection of this misfolded protein is the only known means by which a prion disease can be diagnosed, and it is typically performed after death. The authors developed an approach for the detection of this misfolded protein using a technique known as real-time quaking-induced conversion (RT-QuIC) from secretions and excretions. Real-time quaking-induced conversion is a powerful tool to detect infectious fecal prions from CWD-infected white-tailed deer, well before the onset of clinical disease.

Interestingly, Peng et al., developed an intelligent genotyping platform, PmGT, for *P. multocida* strains according to their whole genome sequences using web 2.0 technology. Using PmGT, the authors determined capsular genotypes, LPS genotypes, and MLST genotypes as well as the main virulence factor genes of *P. multocida* isolates from different host species based on their whole genome sequences published on NCBI. The results revealed a close association between the genotypes and pasteurellosis rather than between genotypes and host species. With the advent of high-quality, inexpensive DNA sequencing platforms, PmGT represents a more efficient tool for *P. multocida* diagnosis in both epidemiological studies and clinical settings.

Finally, a review article by Cameron et al., was included in our Research Topic section, covering the current knowledge and limitations of the use of field-based nucleic acid amplification technology termed loop-mediated isothermal amplification (LAMP) for the diagnosis of honey bee pathogens and pests. Loop-mediated isothermal amplification assays have become an important tool in the last few years in the detection of both exotic and endemic pathogens in the livestock industry.

Taken together, all the published papers in this Research Topic contribute to the development and improvement of techniques and strategies to control animal infectious diseases, exploring novel diagnostic tests, and new technologies that have the potential both to advance our understanding of bacterial/viral animal infections and to improve current diagnostic and control strategies.

## Author Contributions

All authors listed as Guest Editor for this Research Topic have made a substantial, direct, and intellectual contribution to the work and approved it for publication.

## Conflict of Interest

The authors declare that the research was conducted in the absence of any commercial or financial relationships that could be construed as a potential conflict of interest.

## Publisher's Note

All claims expressed in this article are solely those of the authors and do not necessarily represent those of their affiliated organizations, or those of the publisher, the editors and the reviewers. Any product that may be evaluated in this article, or claim that may be made by its manufacturer, is not guaranteed or endorsed by the publisher.

